# Long-Term Stroke Risk in Patients With New Ischemic Brain Lesions on MRI After Carotid Revascularization

**DOI:** 10.1161/STROKEAHA.123.043336

**Published:** 2023-08-24

**Authors:** Simone J.A. Donners, Marjolijn L. Rots, Raechel J. Toorop, Aad van der Lugt, Leo H. Bonati, Gert J. de Borst

**Affiliations:** Department of Vascular Surgery, University Medical Center Utrecht, the Netherlands (S.J.A.D., R.J.T., G.J.d.B.).; Department of General Surgery, Jeroen Bosch Hospital, Den Bosch, the Netherlands (M.L.R.).; Department of Radiology and Nuclear Medicine, Erasmus Medical Centre, Rotterdam, the Netherlands (A.v.d.L.).; Department of Neurology and Stroke Center, University Hospital Basel, Switzerland (L.H.B.).

**Keywords:** carotid endarterectomy, carotid stenosis, diffusion-weighted imaging, lesion, stroke

## Abstract

**BACKGROUND:**

Carotid artery revascularization can result in new ischemic brain lesions on diffusion-weighted magnetic resonance imaging. This study aimed to investigate the relationship between periprocedural ischemic diffusion-weighted imaging (DWI) lesions after carotid artery revascularization and recurrent long-term cerebrovascular events.

**METHODS:**

A secondary observational prospective cohort analysis of existing clinical trial data was performed on 162 patients with symptomatic carotid stenosis that were previously randomized to carotid artery stenting or carotid endarterectomy in the ICSS (International Carotid Stenting Study) and included in the magnetic resonance imaging substudy. Magnetic resonance imagings were performed 1 to 7 days before and 1 to 3 days after treatment. The primary composite clinical outcome was the time to any stroke or transient ischemic attack during follow-up. Patients with new diffusion-weighted imaging (DWI) lesions on posttreatment magnetic resonance imaging scan (DWI+) were compared with patients without new lesions (DWI–).

**RESULTS:**

The median time of follow-up was 8.6 years (interquartile range, 5.0–12.5). Kaplan-Meier cumulative incidence for the primary outcome after 12.5-year follow-up was 35.3% (SE, 8.9%) in DWI+ patients and 31.1% (SE, 5.6%) in DWI− patients. Uni- and multivariable regression analyses did not show significant differences (hazard ratio, 1.50 [95% CI, 0.76–2.94] and hazard ratio, 1.30 [95% CI, 0.10–1.02], respectively). Higher event rate of the primary outcome in DWI+ patients in the overall cohort was mainly caused by events in the carotid artery stenting group.

**CONCLUSIONS:**

Based on our outcome analysis within the ICSS magnetic resonance imaging substudy, DWI lesions following carotid revascularization did not seem to have a relationship with long-term stroke risk.

**REGISTRATION:**

URL: https://www.clinicaltrials.gov; Unique identifier: ISRCTN 25337470.

In symptomatic patients with atherosclerotic stenosis of the extracranial carotid artery, revascularization procedures can result in new ischemic brain lesions.^1,2^ The majority of diffusion-weighted imaging (DWI) lesions on magnetic resonance imaging (MRI) are not associated with neurological symptoms, whereas sometimes DWI lesions leave permanent tissue damage visible on fluid-attenuated inversion recovery (FLAIR) sequences.^3^ Since the classic primary clinical outcomes (stroke and cardiovascular death) occur at low rates after carotid artery stenting (CAS) and carotid endarterectomy (CEA), a surrogate outcome marker may be justified. DWI lesions are procedure-related and occur more often than periprocedural stroke and have therefore increasingly been proposed as a surrogate marker for stroke.^4^

The ICSS (International Carotid Stenting Study) randomized patients with symptomatic carotid stenosis to CAS or CEA.^5^ The MRI substudy of the ICSS demonstrated that DWI lesions on MR-DWI were present more often after CAS (50%) than after CEA (17%).^3^ A 5-year follow-up study established the clinical relevance of this surrogate marker by showing that patients with new DWI lesions after CAS had an increased chance of recurrent stroke or transient ischemic attack (TIA) compared with those without (hazard ratio [HR], 2.85 [95% CI, 1.05–7.72]; *P*=0.040).^6^ However, this study failed to demonstrate the association between DWI lesions and the risk of recurrent stroke or TIA after a CEA procedure, possibly due to the low number of DWI lesions and recurrent events.

The establishment of the longer-term clinical relevance of the DWI lesions after carotid revascularization procedures is essential in the process of implementing a universally accepted surrogate outcome. Today, thus far, there are no data describing adverse events in DWI-positive patients beyond 5-year follow-up. Therefore, in this study,we intended to determine the long-term cerebrovascular outcome in DWI-positive compared with DWI-negative patients after carotid revascularization procedures.

## METHODS

### Study Design and Participants

All patients of the prospective multicenter ICSS MRI substudy recruited by the University Medical Center Utrecht (UMCU) and Erasmus Medical Center (Erasmus MC) Rotterdam were eligible for the current study. The study design and the short- and long-term results have been published previously.^3,6,7^ In brief, symptomatic patients (symptoms attributable to the ipsilateral artery within 12 months before randomization consisting of retinal ischemia, TIA, or hemispheric stroke) with a carotid stenosis ≥50% according to the North American Symptomatic CEA Trial method^8^ were randomly allocated in a 1:1 ratio to CEA or CAS. Patients who had a major stroke, previous revascularization in the ipsilateral artery, contraindications for either treatment, or who were planned for major surgery were excluded. The use of a cerebral protection device during CAS was not mandatory but was recommended in case the device could be safely deployed. All patients received antiplatelet therapy, or anticoagulation if indicated, and a combination of aspirin and clopidogrel to cover CAS procedures was recommended. Follow-up in UMCU and Erasmus MC in the context of the original ICSS study was 5 years. For the current study, a written request letter for information was sent to the general practitioner by the treating specialist of the patient to register any adverse events in the successive years to acquire complete 12.5-year follow-up. The occurrence of cerebrovascular events (including the side) and death was requested, with the dates of the events that may have occurred. In case of no response, a second letter and later a reminder by telephone was given by the treating physician. The obtained data were subsequently added to the available database with earlier collected data. The data that support the findings of this study are available from the corresponding author upon reasonable request. The ICSS MRI substudy was approved by the Medical Research Ethics Committee of University Medical Center Utrecht. Patients provided written informed consent. This study complies with the STROBE (Strengthening the Reporting of Observational Studies in Epidemiology) guidelines^9^ (see STOBE checklist in the Supplemental Material).

### Magnetic Resonance Imaging

In the course of the study protocol of the ICSS MRI substudy, patients underwent MRI scans at field strengths of 1.5 or 3 Tesla 1 to 7 days before treatment (pretreatment scan) and 1 to 3 days after treatment (posttreatment scan) using DWI and FLAIR sequences. MRI scans were assessed by an experienced neurologist and neuroradiologist, both masked to treatment. If disagreement could not be resolved by consensus, a third reviewer made the final decision. The presence of new periprocedural ischemic brain lesions was defined as hyperintense DWI lesions on the post-treatment MRI that were not present on the pretreatment MRI. Also, the number (lesion count) of new DWI lesions was assessed in each patient. Baseline white matter hyperintensities were quantified on pretreatment FLAIR sequences with the age-related-white matter changes (ARWMC) score.^10^

### Clinical Outcome

The primary composite clinical outcome consisted of stroke or TIA located in any vascular territory, occurring between the post-treatment MRI scan and the end of available follow-up. Stroke was defined as a rapidly developing clinical syndrome of focal disturbance of cerebral function lasting >24 hours or leading to death with no apparent cause other than that of vascular origin. TIA was defined as a rapidly developing clinical syndrome of focal disturbance of cerebral function lasting <24 hour with no apparent cause other than cerebral ischemia. Secondary outcomes were ipsilateral stroke/TIA, any stroke, and ipsilateral stroke.

### Statistical Analysis

Quantitative data were summarized as median (interquartile range) and were compared using the Mann-Whitney *U* test. Discrete data were presented as frequencies and percentages and were compared using Pearson Chi-squared test or Fisher exact test as appropriate. Demographic and clinical baseline characteristics were compared between patients with (DWI+) and without new DWI lesions (DWI−) after treatment. Data were inspected for patterns of missing values. The proportion of randomly missing values for baseline characteristics did not exceed 1%. The cumulative incidence of cerebrovascular events of DWI+ versus DWI− patients were estimated with Kaplan-Meier statistics and their SEs at 12.5 years after the posttreatment MRI scan. Mortality in both groups was compared by performing the log-rank test. The association of DWI presence with cerebrovascular events was analyzed in 2 ways: (1) DWI+ compared with DWI− in binary analysis (using DWI− as reference group) and (2) DWI count as a continuous quantification analysis. Cox proportional hazard regression models were used to calculate the hazard ratio (HR) with 95% CI for the association between DWI presence and cerebrovascular events during follow-up. The proportional hazard assumption was verified by examining the Schoenfeld residuals. Models were adjusted for the demographic or clinical variables that showed an association of *P*<0.20 with the determinant (DWI presence) as well as with the outcome of interest (Table S1). Cox regressions of DWI count were checked for sampling distribution by bootstrapping HR CIs with 100 000 iterations using the bias-corrected method with R statistical software (package boot.pval). In the case of the occurrence of multiple events in follow-up, only the first event qualifying as an outcome event was counted for analysis. A *P*<0.05 was considered statistically significant. All statistical analyses were performed with R version 4.2.1 (R Foundation for Statistical Computing, Vienna, Austria), inside an R Studio 2022.07.2 environment.

## RESULTS

The present analysis included all 162 patients of the UMCU and Erasmus MC that were randomized in the ICSS-MRI substudy between January 2004 and October 2008, of whom 110 GPs responded and provided long-term follow-up data for the present study (Figure S1). Patients in the DWI+ group were more often treated with CAS (*P*=0.002; Table 1). Total cholesterol at randomization was higher in the DWI− group (*P*=0.043). In the group randomized to CAS, 25 patients were treated with cerebral protection devices.

**Table 1.**
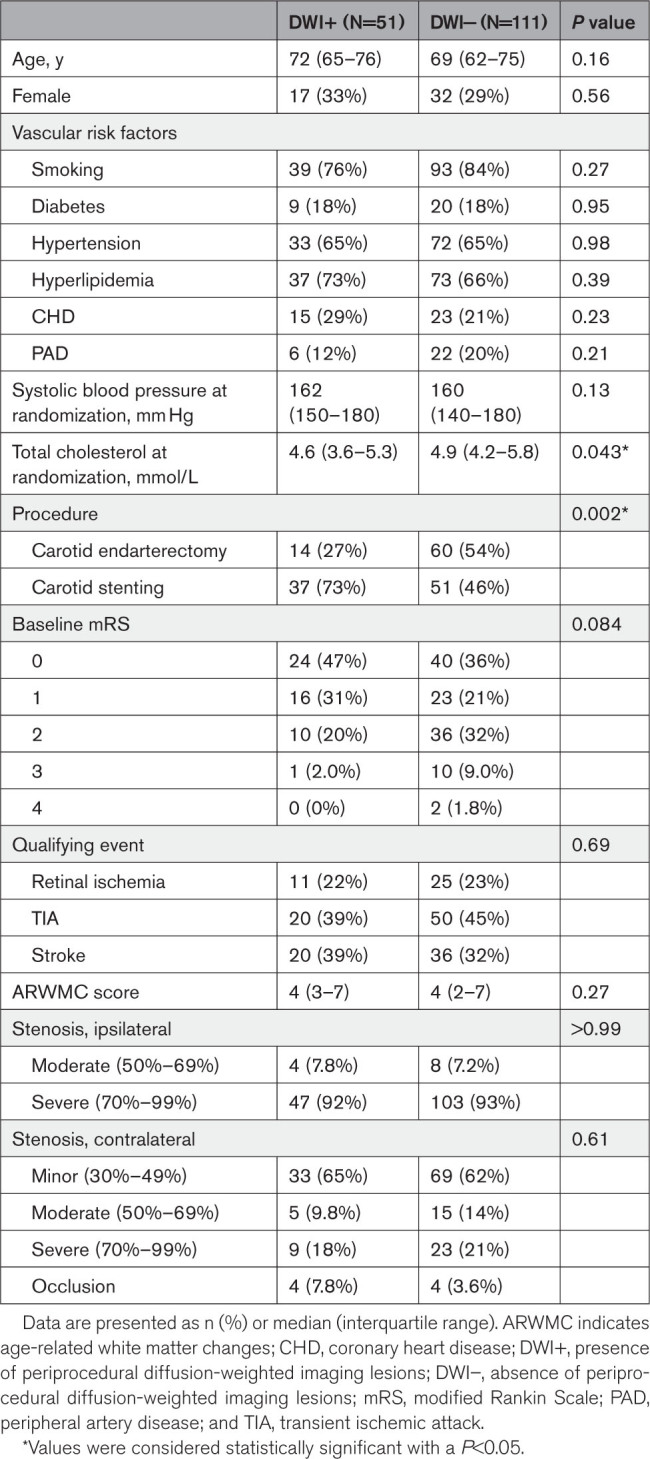
Baseline Characteristics of Patients With and Without New Periprocedural DWI Lesions

**Table 2.**
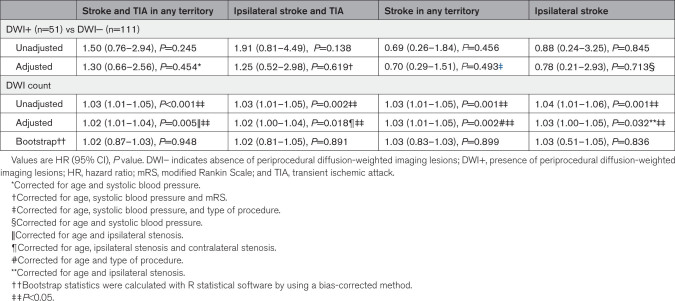
HRs of Outcome Events During Follow-Up Depending on New Postoperative DWI Lesions

In the total cohort, 51 (31.5%) patients had the presence of new periprocedural DWI lesions on posttreatment MRI. During a median follow-up of 8.6 years (interquartile range, 5.0–12.5), 19 patients died in the DWI+ group (cumulative 12.5-year mortality rate, 46.0%; SE, 5.5%) and 40 patients died in the DWI− group (51.3%; SE, 9.0%). Thirty-seven (22.8%) patients reached the primary outcome of recurrent stroke or TIA in any territory. Kaplan-Meier cumulative incidence for the primary outcome after 12.5-year follow-up was 35.3% (SE, 8.9%) in DWI+ patients and 31.1% (5.6%) in DWI− patients (Figure). The binary uni- and multivariable cox regression analyses revealed no significant difference in recurrent cerebrovascular events between DWI+ patients and DWI− patients (Table 2). Given the high mortality risk in the study population, sensitivity analyses were performed by conducting Fine-Gray competing risk modeling with R statistical software (package cmprsk), which showed similar results (Table S2). In the continued analysis, DWI lesion count was positively associated with all 4 clinical outcomes. However, after bootstrapping the HR CIs, these results no longer remained significant.

**Figure.**
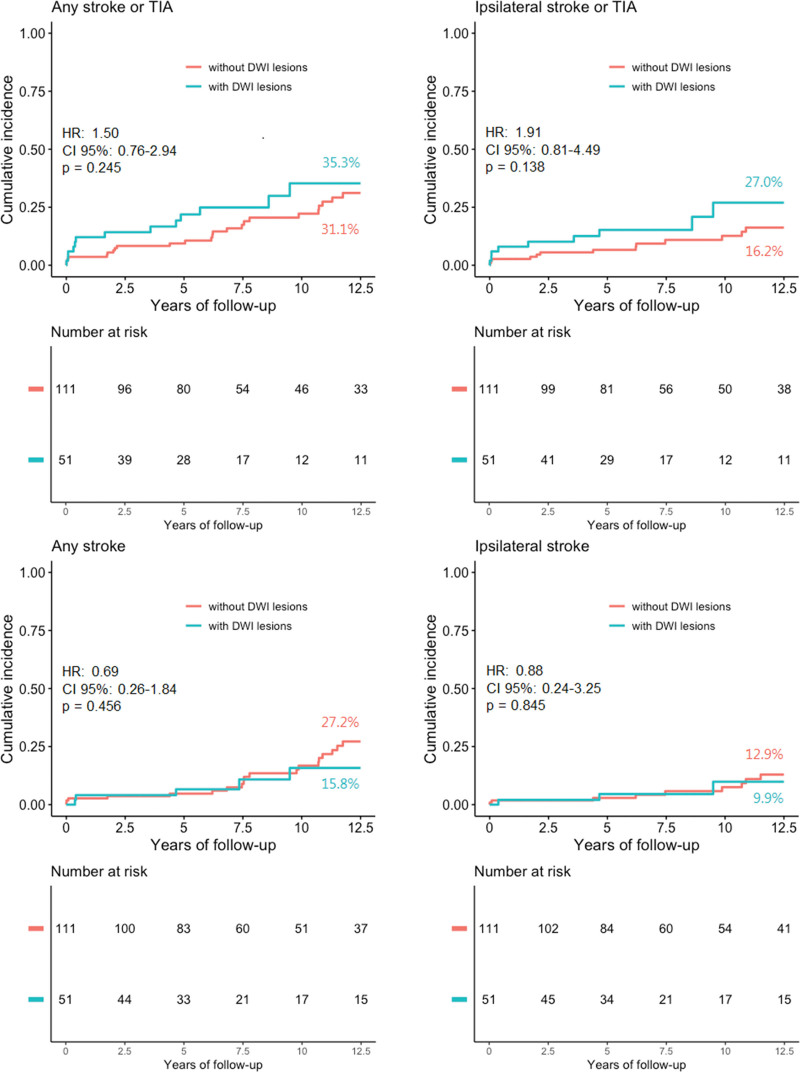
**Kaplan-Meier curves of outcome events during 12.5-y follow-up.** Comparison of any stroke or transient ischemic attack (TIA), ipsilateral stroke or TIA, any stroke and ipsilateral stroke between patients with postprocedural diffusion-weighted imaging (DWI) lesions (green curves) and without postprocedural DWI lesions (orange curve) after carotid revascularization. Percentages are point estimates of cumulative incidences after 12.5 y of follow-up. HR indicates hazard ratio.

Separate analyses for the CAS and CEA were performed (Supplemental Material). In the CAS group, 51 patients were DWI+ versus 37 DWI−. Patients with DWI lesions were older and had lower total cholesterol at randomization (Table S3). In the CEA group, 14 patients were DWI+ versus 60 patients DWI−, without significant differences at baseline. Based on the results of the Kaplan-Meier curves, the higher event rate of the primary outcome in DWI+ patients in the overall cohort was mainly caused by the events in the CAS group (Figure S2A through S2H). Univariate analyses were inaccurate (wide CI) and multivariable analyses could not be performed, both because of limited power.

## DISCUSSION

The current study demonstrated that new periprocedural DWI lesions were not associated with a higher risk of recurrent stroke or TIA at long-term follow-up in symptomatic patients after carotid revascularization with CEA or CAS.

In the ICSS MRI substudy, both the presence and count of DWI lesions were associated with recurrent stroke/TIA occurring after the posttreatment scan with most events seen in the first 6 months after treatment.^6^ The results of the present study showed that this association was no longer present after 12.5 years of follow-up. In addition, the analyses with DWI counts were largely influenced by the small number of patients with high DWI lesions, which lost the association with the clinical outcomes after bootstrapping the HR CIs. This supports the hypothesis that DWI lesions are caused by thromboembolic material or atherosclerotic plaque debris during and after the carotid revascularization procedure and therefore might be used as surrogate markers of early recurrent stroke/TIA but not for long-term ischemic events. The hemodynamic disturbance may also play a role in the development of DWI lesions as a result of impaired clearance of emboli.^11^ A recent study showed that the absence of the anterior communicating artery in the circle of Willis on MRI was associated with reduced cerebrovascular reactivity in the ipsilateral middle cerebral artery territory and oxygen saturation after temporary clamping of the carotid artery during carotid revascularization. Also, an increased incidence of new ischemic lesions on DWI was seen.^12^

Currently, DWI lesions are already being used as periprocedural outcome marker in, for example, carotid stenting studies. A randomized controlled trial comparing conventional single-layer versus MicroNet-covered stents showed that the latter significantly reduced the amount and volume of periprocedural ipsilateral DWI lesions, and a 3:1 difference was seen in the number of permanent lesions on FLAIR after 30 days.^13^ Another retrospective study showed the presence of DWI lesions in 8 of 110 (7.3%) patients 24 hours after stenting with a double layer stent combined with a distal embolic protection device, which is remarkably lower compared with other conventional stenting studies.^14^

This study failed to demonstrate the relevance of DWI lesions after CEA and CAS separately, due to the small sample sizes and number of events. Outcome events were mainly caused by DWI+ patients in the stenting group, which may be explained by the higher age in this group. A meta-analysis in 2017 showed a stable ratio when comparing new DWI lesions to the number of strokes and TIAs among patients undergoing different carotid artery intervention (CEA and CAS).^15^ Therefore, it was assumed that CEA and CAS could be analyzed simultaneously in the present study. However, previous results of the ICSS MRI substudy revealed that the development of DWI lesions differed between CEA and CAS, showing that lesions of CAS were more frequently present but were less likely to progress from acute to persisting lesions on FLAIR imaging 1 month after treatment.^16^ Therefore, assessing the possibility of DWI lesions as a periprocedural surrogate marker for stroke can still be considered for CEA and CAS separately in the future.

Increasing attention has arisen toward the concomitant risks of the management of the carotid disease on cognition. Irreversible (silent) brain infarcts are already proposed as predictor for cognitive decline.^17,18^ However, evidence for causality between DWI lesions and cognitive decline is still pending. The NeuroVISION study, including 1114 noncardiac surgery patients (no CEA patients) of which 7% developed DWI lesions, reported that 42% DWI+ patients developed cognitive impairment after 1 year compared with 29% DWI− patients (adjusted OR, 1.98 [95% CI, 1.22–3.20]; *P*=0.006).^19^ Also, other small cohort studies showed short-term impaired cognitive function in DWI+ patients after revascularization procedures^20–22^; however, studies with long-term follow-up data are still lacking. We speculate that only DWI lesions that progress to a chronic stage lead to permanent brain damage and thus DWI lesions represent a preliminary stage of brain damage. However, this should be investigated in future MRI studies with long-term cognitive evaluation.

Important to note is that definition remains key in studying the relevance of new periprocedural DWI lesions.^23^ Presence of silent brain infarction on MRI (consisting of areas of focal hyperintensity on T2 and hypointensity on T1-weighted images) is already strongly associated with future symptomatic stroke in multiple studies.^24–26^ If one wishes to explore the ability of DWI lesions as a surrogate marker for periprocedural stroke, the assessment can use diffusion-restricted brain areas with MR-DWI, with even higher sensitivity when appearing hypo-/isointense on apparent diffusion coefficient.

The strengths of our study relate to the use of data from a multicenter randomized controlled trial. This study also has some limitations. First, both 1.5- and 3-T MRI scanners were used, which may have caused a difference in sensitivity in the detection of DWI lesions. Second, its retrospective nature and small sample size since only patients from UMCU and Erasmus MC were included. Moreover, some GPs did not respond or could not be retrieved. Third, in the first 5 years, the follow-up data were retrieved annually with event adjudication by an independent outcome committee. In the current study, the successive years were requested through the general practitioner with the risk that events may have been missed or misattributed. Also, the previously published ICSS MRI substudy^6^ stated that none of the patients with recurrent events underwent CAS or CEA, but in the current study, this information was not available for the consecutive years. Last, there is no information on potentially started add-on therapies such as lipid-lowering PCSK-9 inhibitors and anti-inflammatory colchicine, both of which show promising results in reducing cardiovascular events.^27,28^

In conclusion, the results from our analysis do not support the role of ischemic brain lesions discovered on DWI after carotid revascularization procedures as risk markers for long-term recurrent stroke or TIA. However, as new periprocedural DWI lesions seem to be a marker for early recurrent cerebrovascular events, future randomized studies are needed to evaluate whether the effect of treatment on these lesions corresponds to the effect of treatment on procedural stroke before surrogacy can be validated.

## ARTICLE INFORMATION

### Sources of Funding

This research did not receive any specific grant from funding agencies in the public, commercial, or not-for-profit sectors.

### Disclosures

Dr van der Lugt received research grants from GE Healthcare, Siemens Healthineers, Cerenovus, Medtronic USA, Inc and Stryker Corporation, and Philips Healthcare all paid to the institution. Dr Bonati has received grants from Swiss National Science Foundation, University of Basel, Swiss Heart Foundation, AstraZeneca and Stiftung zur Förderung der gastroenterologischen und allgemeinen klinischen Forschung sowie der medizinischen Bildauswertung; personal fees from Amgen, Bayer, Bristol-Myers Squibb, Claret Medical, and InnovHeart; and nonfinancial support from AstraZeneca and Bayer. All other authors have reported that they have no relationships relevant to the contents of this article to disclose.

### Supplemental Material

Tables S1–S3

Figures S1–S2

## Supplementary Material


