# Supplementary material for: Long-Term Stroke Risk in Patients With New Ischemic Brain Lesions on MRI After Carotid Revascularization
Source: Stroke. 2023 Aug 24;54(10):2562–8. doi: 10.1161/STROKEAHA.123.043336 (PMC10519293; doi:10.1161/STROKEAHA.123.043336)
Supplement: Supplementary file 1 [file str-54-2562-s001.pdf]

STROBE Statement—checklist of items that should be included in reports of observational studies

	Item No	Recommendation	Location within manuscript
**Title and abstract**	1	(*a*) Indicate the study's design with a commonly used term in the title or the abstract	Page 2 Methods
		(*b*) Provide in the abstract an informative and balanced summary of what was done and what was found	Page 2 Methods and conclusion
**Introduction**			
Background/rationale	2	Explain the scientific background and rationale for the investigation being reported	Page 4 Introduction,
Objectives	3	State specific objectives, including any prespecified hypotheses	Page 4 Introduction, last paragraph
**Methods**			
Study design	4	Present key elements of study design early in the paper	Page 5 Study design and participants
Setting	5	Describe the setting, locations, and relevant dates, including periods of recruitment, exposure, follow-up, and data collection	Page 5 Study design and participants
Participants	6	(*a*) *Cohort study*—Give the eligibility criteria, and the sources and methods of selection of participants. Describe methods of follow-up	Page 5 Study design and participants
Variables	7	Clearly define all outcomes, exposures, predictors, potential confounders, and effect modifiers. Give diagnostic criteria, if applicable	Page 6 Outcome and Statistical analysis
Data sources/ measurement	8*	For each variable of interest, give sources of data and details of methods of assessment (measurement). Describe comparability of assessment methods if there is more than one group	Page 5 and 6 MRI, clinical outcome
Bias	9	Describe any efforts to address potential sources of bias	Page 5 Study design and participants
Study size	10	Explain how the study size was arrived at	Page 5 Study design and participants
Quantitative variables	11	Explain how quantitative variables were handled in the analyses. If applicable, describe which groupings were chosen and why	Page 6 Clinical outcome and Statistical analysis
Statistical methods	12	(*a*) Describe all statistical methods, including those used to control for confounding	Page 6 Statistical analysis
		(*b*) Describe any methods used to examine subgroups and interactions	Page 8 Results
		(*c*) Explain how missing data were addressed	Page 6 Statistical analysis
		(*d*) *Cohort study*—If applicable, explain how loss to follow-up was addressed	Page 5 Study design and participants
		(*e*) Describe any sensitivity analyses	N/A

Results **Location within**

			manuscript
Participants	13*	(a) Report numbers of individuals at each stage of study—eg numbers potentially eligible, examined for eligibility, confirmed eligible, included in the study, completing follow-up, and analysed	Page 6 Results
		(b) Give reasons for non-participation at each stage	Page 5 Study design and participants
		(c) Consider use of a flow diagram	N/A
Descriptive data	14*	(a) Give characteristics of study participants (eg demographic, clinical, social) and information on exposures and potential confounders	Page 8 Results
		(b) Indicate number of participants with missing data for each variable of interest	Figure 1
		(c) *Cohort study*—Summarise follow-up time (eg, average and total amount)	Page 8 Results
Outcome data	15*	*Cohort study*—Report numbers of outcome events or summary measures over time	Page 8 Results
Main results	16	(*a*) Give unadjusted estimates and, if applicable, confounder-adjusted estimates and their precision (eg, 95% confidence interval). Make clear which confounders were adjusted for and why they were included	Page 6 Statistical analysis, Page 8 Results and supplemental material.
		(*b*) Report category boundaries when continuous variables were categorized	N/A
		(*c*) If relevant, consider translating estimates of relative risk into absolute risk for a meaningful time period	N/A
Other analyses	17	Report other analyses done—eg analyses of subgroups and interactions, and sensitivity analyses	Page 8 Results
**Discussion**			
Key results	18	Summarise key results with reference to study objectives	Page 9 Discussion first paragraph
Limitations	19	Discuss limitations of the study, taking into account sources of potential bias or imprecision. Discuss both direction and magnitude of any potential bias	Page 10/11 Discussion
Interpretation	20	Give a cautious overall interpretation of results considering objectives, limitations, multiplicity of analyses, results from similar studies, and other relevant evidence	Page 11 Discussion last paragraph
Generalisability	21	Discuss the generalisability (external validity) of the study results	Page 9 Discussion
**Other information**			
Funding	22	Give the source of funding and the role of the funders for the present study and, if applicable, for the original study on which the present article is based	N/A
