# Supplementary material for: Long-Term Stroke Risk in Patients With New Ischemic Brain Lesions on MRI After Carotid Revascularization
Source: Stroke. 2023 Aug 24;54(10):2562–8. doi: 10.1161/STROKEAHA.123.043336 (PMC10519293; doi:10.1161/STROKEAHA.123.043336)
Supplement: Supplementary file 2 [file str-54-2562-s002.pdf]

SUPPLEMENTAL MATERIAL

<u>Contents</u>

Supplemental Table S1. Univariate associations between baseline characteristics and cerebrovascular events during follow-up

Supplemental Figure S1. Study flow chart

Supplemental Table S2. Results of the sensitivity analysis estimated by Fine-Gray competing risk modeling

Supplemental Table S3. Baseline characteristics of patients with and without periprocedural DWI lesions in the carotid stenting and carotid endarterectomy group

Supplemental Figure S2. Kaplan-Meier curves with univariable cox regressions carotid stenting (A,B,C,D) and carotid endarterectomy (E,F,G,H)

Table S1. Univariate associations between baseline characteristics and cerebrovascular events during follow-up

	Stroke or TIA in any territory	Ipsilateral stroke or TIA	Stroke in any territory	Ipsilateral stroke in any territory
Age	**1.06 (1.02, 1.10), 0.004**	**1.03 (0.98, 1.09), 0.191**	**1.09 (1.03, 1.14), <0.001**	**1.08 (1.01, 1.16), 0.021**
Female sex	1.25 (0.64, 2.46), 0.516	1.06 (0.43, 2.61), 0.895	1.29 (0.56, 2.94), 0.557	1.53 (0.48, 4.82), 0.477
Smoking	0.95 (0.42, 2.17), 0.911	1.04 (0.35, 3.07), 0.946	1.17 (0.40, 3.42), 0.774	0.45 (0.14, 1.51), 0.222
Diabetes	**1.80 (0.85, 3.83), 0.145**	**2.16 (0.84, 5.54), 0.133**	**2.46 (1.02, 5.94), 0.063**	1.90 (0.51, 7.01), 0.366
Hypertension	0.99 (0.51, 1.93), 0.983	1.08 (0.45, 2.59), 0.855	1.63 (0.68, 3.94), 0.262	1.29 (0.39, 4.29), 0.673
Hyperlipidaemia	0.96 0.49, 1.88), 0.896	1.38 (0.54, 3.52), 0.496	0.84 (0.37, 1.92), 0.683	1.58 (0.43, 5.85), 0.475
CHD	1.31 (0.63, 2.70), 0.481	1.72 (0.70, 4.22), 0.255	1.19 (0.47, 3.00), 0.716	1.82 (0.55, 6.05), 0.348
PAD	1.40 (0.61, 3.20), 0.441	1.71 (0.63, 4.65), 0.320	1.76 (0.65, 4.74), 0.292	1.18 (0.26, 5.42), 0.831
Systolic blood pressure*	**1.01 (1.00, 1.02), 0.077**	**1.02 (1.01, 1.04), 0.007**	**1.02 (1.00, 1.03), 0.031**	**1.03 (1.01, 1.06), 0.004**
Total cholesterol*	0.95 (0.74, 1.24), 0.722	1.10 (0.80, 1.51), 0.556	1.09 (0.80, 1.50), 0.591	1.27 (0.85, 1.88), 0.258
Procedure	1.01 (0.53, 1.93), 0.971	1.04 (0.45, 2.40), 0.934	**0.59 (0.26, 1.33), 0.198**	0.60 (0.19, 1.90), 0.382
mRS*	1.01 (0.46, 2.22), 0.872	**1.19 (0.48, 2.96), 0.155**	0.56 (0.18, 1.75), 0.576	0.68 (0.18, 2.62), 0.599
Stroke as qualifying event	0.81 (0.33, 2.01), 0.824	0.40 (0.12, 1.37), 0.290	0.83 (0.27, 2.57), 0.904	0.80 (0.16, 3.98), 0.940
ARWMC	**1.07 (0.99, 1.15), 0.109**	1.04 (0.94, 1.14) 0.498	**1.11 (1.01, 1.21), 0.036**	**1.12 (0.99, 1.27), 0.103**
Ipsilateral stenosis†	**0.42 (0.18, 1.02), 0.082**	**0.40 (0.14, 1.19), 0.135**	0.49 (0.17, 1.44), 0.233	**0.30 (0.08, 1.12), 0.109**
Contralateral stenosis‡	0.80 (0.28, 2.29), 0.281	**0.82 (0.24, 2.80), 0.189**	0.57 (0.13, 2.47), 0.710	0.48 (0.06, 3.71), 0.389

Data are unadjusted Cox regression hazard ratios,95% confidence intervals in parentheses and p-values. P-values <0.2 are highlighted in bold. Abbreviations: ARWMC, age-related white matter changes; CHD, coronary heart disease; PAD, peripheral artery disease; mRS, modified Rankin Scale. *At randomisation. †Ipsilateral degree of stenosis (50-69%, 70-99%). ‡Contralateral degree of stenosis (0-49%, 50-69%, 70-99%, occlude

Figure S1. Study flow chart

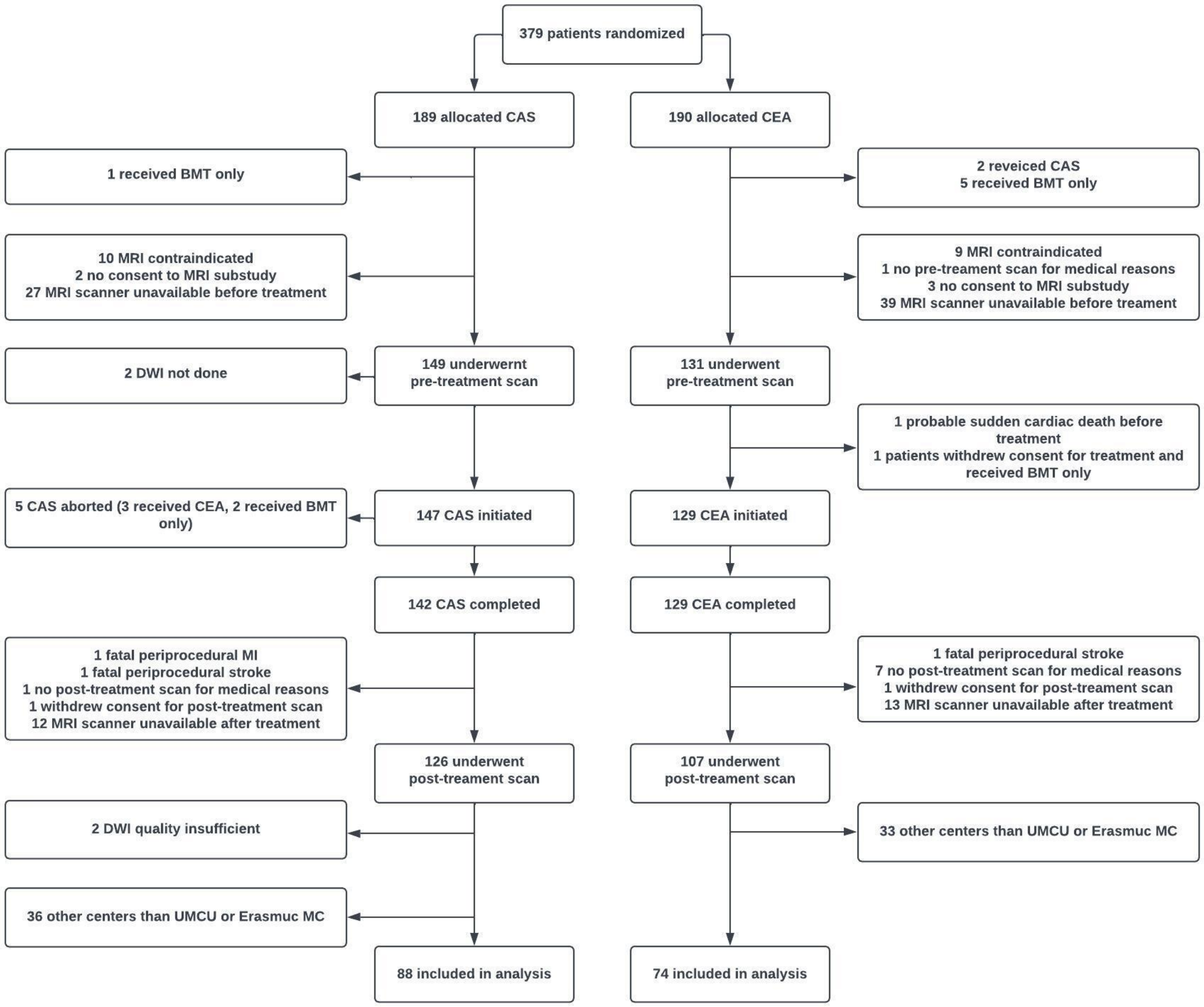

Table S2. Results of the sensitivity analysis estimated by Fine-Gray competing risk modeling

	**Stroke and TIA in any territory**	**Ipsilateral stroke and TIA**	**Stroke in any territory**	**Ipsilateral stroke**
**Unadjusted**	HR 1.35 (0.68-2.66), p=0.4	HR 1.71 (0.74-3.97), p=0.2	HR 0.62 (0.23-1.66), p=0.3	HR 0.77 (0.21-2.84), p=0.7
**Adjusted**	HR 1.16 (0.57-2.36), p=0.7	HR 1.04 (1.00-1.09), p=0.070	HR 0.62 (0.23-1.67), p=0.3	HR 0.62 (0.17-2.31), p=0.5

Table S3. Characteristics of patients with and without periprocedural DWI lesions in the stenting and endarterectomy group

	Stenting			Endarterectomy		
	**DWI+ N = 37**	**DWI− N = 51**	**p-value**	**DWI+ N = 14**	**DWI− N = 60**	**p-value**
**Age, years**	73 (66-78)	67 (60-75)	**0.035**	69 (64-72)	71 (63-75)	0.47
**Female**	13 (35%)	15 (29%)	0.57	4 (29%)	17 (28%)	>0.99
**Smoking**	28 (76%)	42 (82%)	0.44	11 (79%)	51 (85%)	0.69
**Diabetes**	7 (19%)	8 (16%)	0.69	2 (14%)	12 (20%)	>0.99
**Hypertension**	24 (65%)	33 (65%)	0.99	9 (64%)	39 (65%)	>0.99
**Hyperlipidaemia**	27 (73%)	29 (57%)	0.12	10 (71%)	44 (73%)	>0.99
**CHD**	11 (30%)	10 (20%)	0.27	4 (29%)	13 (22%)	0.72
**PAD**	4 (11%)	14 (27%)	0.056	2 (14%)	8 (13%)	>0.99
**Systolic blood pressure at randomization, mm Hg**	160 (148-180)	160 (136-180)	0.43	170 (160-180)	160 (140-178)	0.10
**Total cholesterol at randomization, mmol/L**	4.4 (3.4-5.2)	5.0 (4.2-5.9)	**0.006**	4.8 (4.3-6.0)	4.7 (4.1-5.4)	0.76
**Baseline mRS**			0.26			0.18
0	15 (41%)	22 (43%)		9 (64%)	18 (30%)	
1	13 (35%)	9 (18%)		3 (21%)	14 (23%)	
2	8 (22%)	14 (27%)		2 (14%)	22 (37%)	
3	1 (2.7%)	5 (9.8%)		0 (0%)	5 (8.3%)	
4	0 (0%)	1 (2.0%)		0 (0%)	1 (1.7%)	
**Qualifying event**			0.33			0.47
Retinal Ischemia	7 (19%)	11 (22%)		4 (29%)	14 (23%)	
TIA	12 (32%)	23 (45%)		8 (57%)	27 (45%)	
Stroke	18 (49%)	17 (33%)		2 (14%)	19 (32%)	
**ARWMC score**	4 (3-7)	4 (2-6)	0.16	4 (3-6)	4 (2-8)	0.94
**Stenosis, ipsilateral**			>0.99			>0.99
Moderate (50–69%)	3 (8.1%)	4 (7.8%)		1 (7.1%)	4 (6.7%)	
Severe (70-99%)	34 (92%)	47 (92%)		13 (93%)	56 (93%)	
**Stenosis, contralateral**			0.83			0.74
Minor (30-49%)	22 (59%)	31 (61%)		11 (79%)	38 (63%)	
Moderate (50–69%)	4 (11%)	5 (9.8%)		1 (7.1%)	10 (17%)	
Severe (70-99%)	7 (19%)	12 (24%)		2 (14%)	11 (18%)	
Occlusion	4 (11%)	3 (5.9%)		0 (0%)	1 (1.7%)	

Data are presented as n (%) or median (interquartile range). Bold values were considered statistically significant with a $p<0.05$. Abbreviations: ARWMC, age-related white matter changes; CHD, coronary heart disease; DWI+, presence of periprocedural diffusion-weighted imaging lesions; DWI–, absence of periprocedural diffusion-weighted imaging lesions; mRS, modified Rankin Scale; PAD, peripheral artery disease; TIA, transient ischemic attack.

Figure S2. Kaplan-Meier curves with univariable cox regressions in carotid stenting (A,B,C,D) and carotid endarterectomy (E,F,G,H)

A

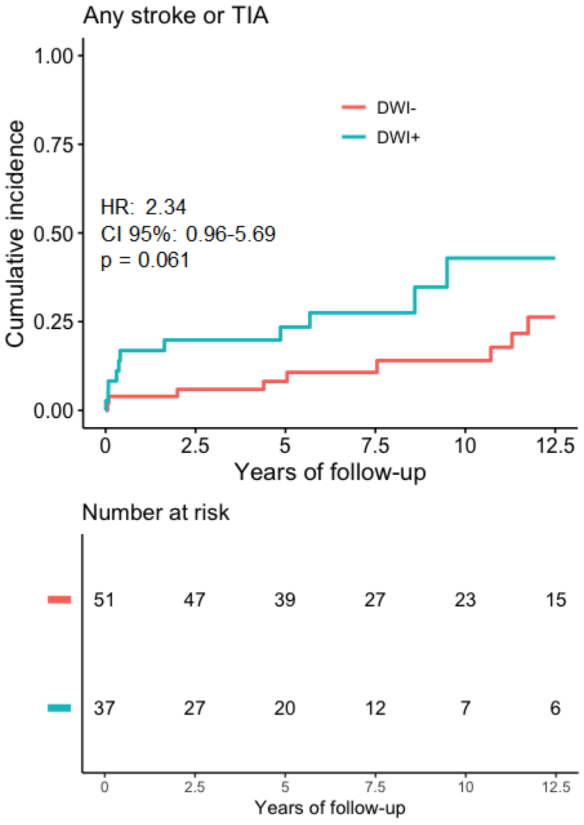

B

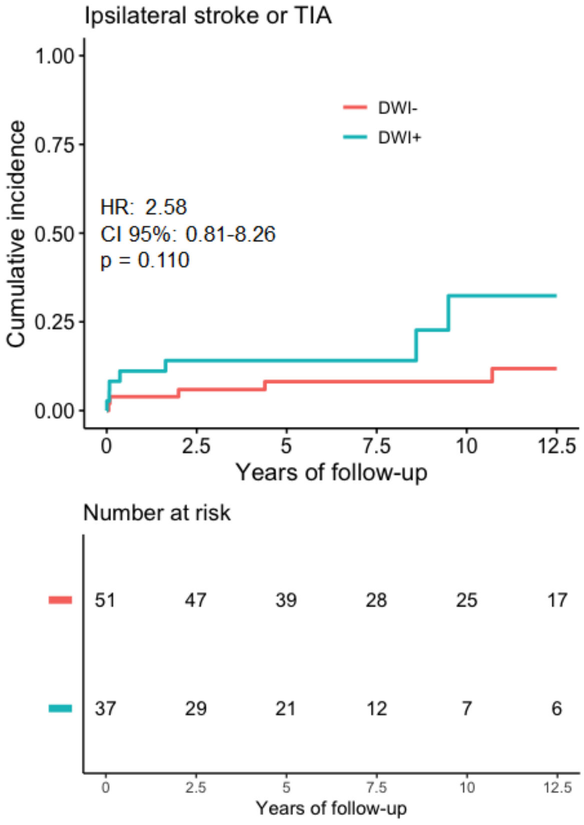

C

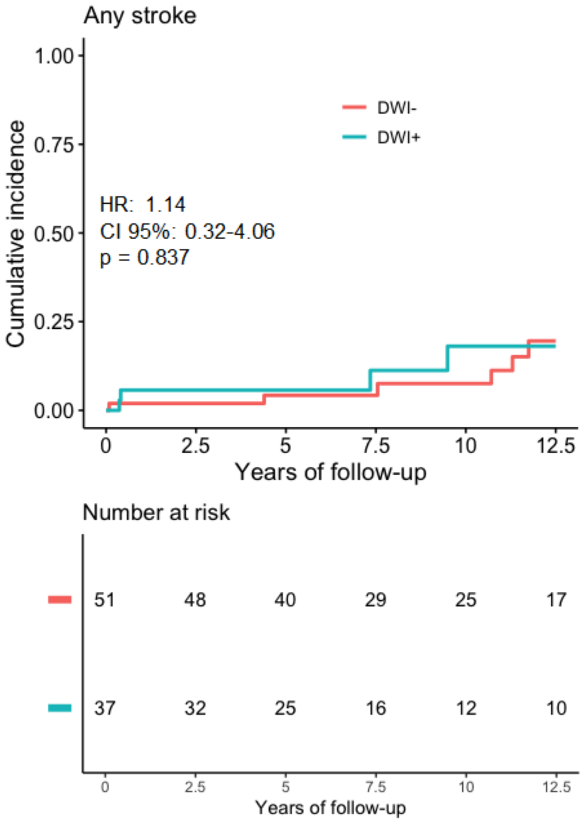

D

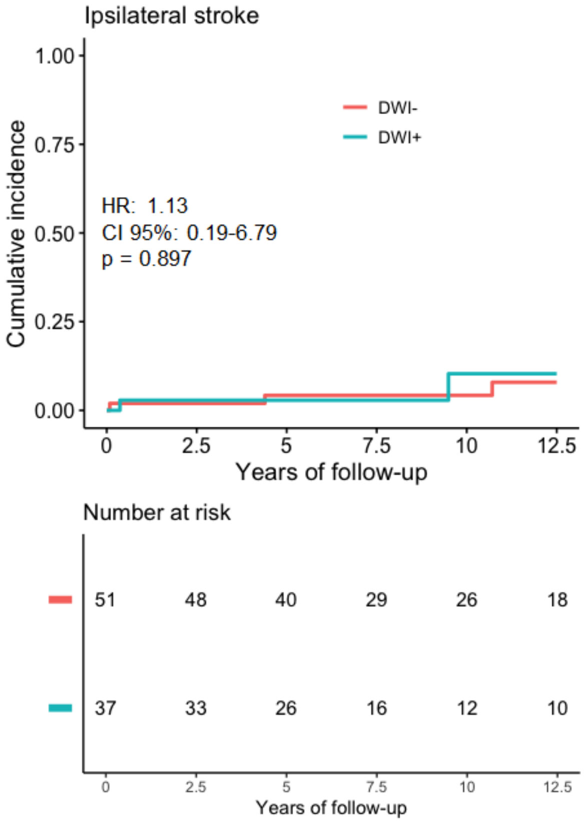

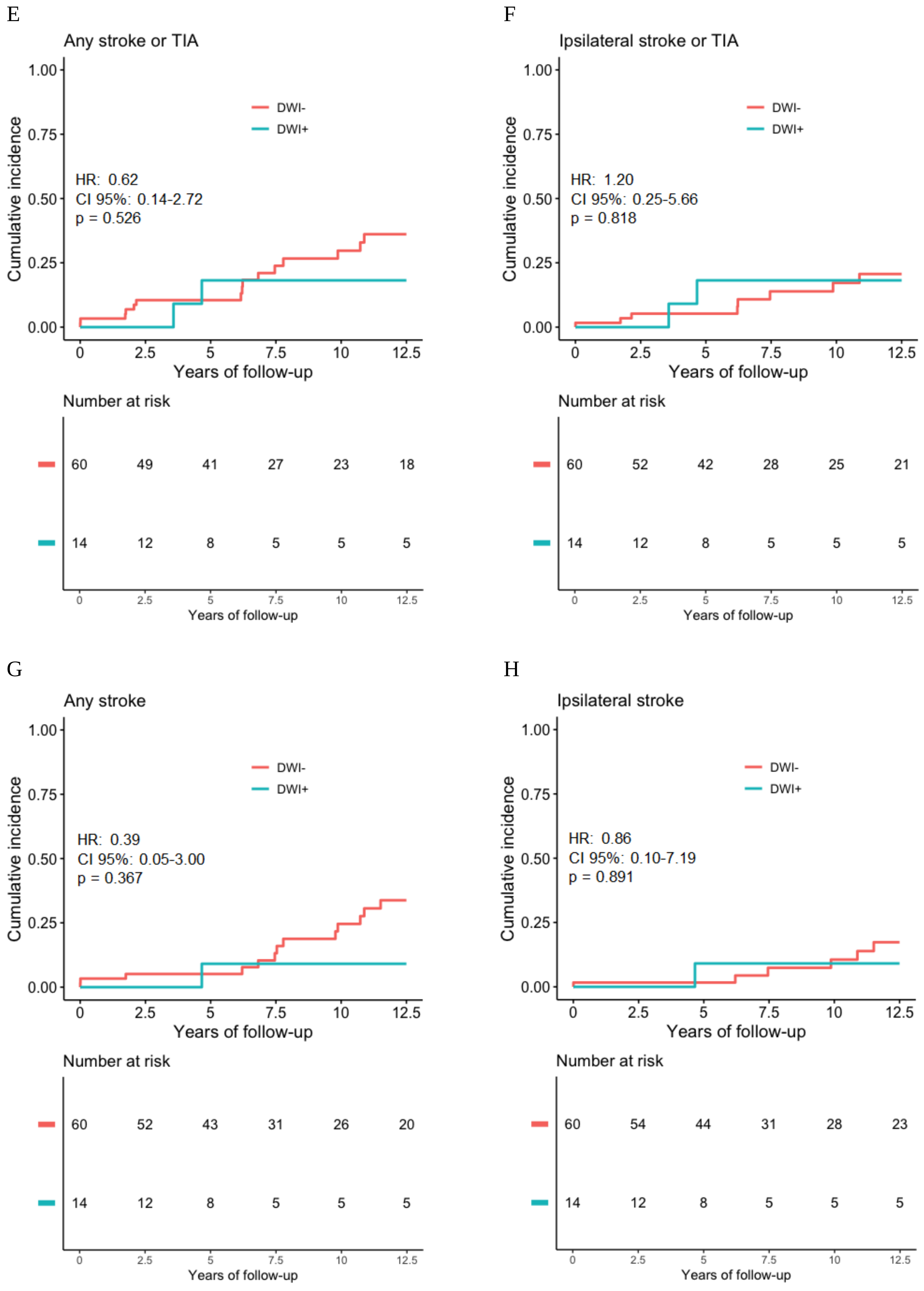
E
Any stroke or TIA
Cumulative incidence
1.00
0.75
0.50
0.25
0.00
DWI-
DWI+
HR: 0.62
CI 95%: 0.14-2.72
p = 0.526
0
2.5
5
7.5
10
12.5
Years of follow-up
Number at risk
60 49 41 27 23 18
14 12 8 5 5 5
Years of follow-up
F
Ipsilateral stroke or TIA
Cumulative incidence
DWI-
DWI+
HR: 1.20
CI 95%: 0.25-5.66
p = 0.818
Years of follow-up
Number at risk
60 52 42 28 25 21
14 12 8 5 5 5
Years of follow-up
G
Any stroke
Cumulative incidence
DWI-
DWI+
HR: 0.39
CI 95%: 0.05-3.00
p = 0.367
Years of follow-up
Number at risk
60 52 43 31 26 20
14 12 8 5 5 5
Years of follow-up
H
Ipsilateral stroke
Cumulative incidence
DWI-
DWI+
HR: 0.86
CI 95%: 0.10-7.19
p = 0.891
Years of follow-up
Number at risk
60 54 44 31 28 23
14 12 8 5 5 5
Years of follow-up
